# Anlotinib dramatically improved pulmonary hypertension and hypoxia caused by Pulmonary Tumor Thrombotic Microangiopathy (PTTM) associated with gastric carcinoma: a case report

**DOI:** 10.1186/s12959-023-00477-4

**Published:** 2023-03-27

**Authors:** Yang Wang, Wei-wei Ning, Yi-fan Jin, Qing-qing Zhu, Zai-liang Wang, Nan Su, Yan-bin Chen, Jian-an Huang, Cheng Chen

**Affiliations:** Pulmonary and Critical Care Medicine, The First Affiliated Hospital of Soochow University, 899 Pinghai Road, Suzhou, 215000 China

**Keywords:** Pulmonary tumor thrombotic microangiopathy, Anlotinib, Pulmonary hypertension, Hypoxia, Gastric carcinoma

## Abstract

**Background:**

Pulmonary tumor thrombotic microangiopathy (PTTM) is a rare malignancy-related respiratory complication, demonstrating rapid progression of pulmonary hypertension (PH) and respiratory failure. Although a number of treatments have been attempted for patients diagnosed with or suspected of having PTTM, successful-treated cases of PTTM were mainly from imatinib therapy, which was a PDGF receptor inhibitor. Anlotinib was a novel tyrosine kinase inhibitor that targets VEGFR, FGFR, PDGFR, and c-kit.

**Case presentation:**

We reported a patient of PTTM associated with gastric carcinoma, whom were treated with anlotinib, thereby exhibiting significant improvement of PH and respiratory dysfunction.

**Conclusion:**

Our case provides a new understanding of therapy to PTTM, with implications for defining anlotinib as candidate drug for PTTM. Clinical diagnosis and prompt initiation of anlotinib might be one of the strategies in patients with unstable PTTM.

## Background

Pulmonary tumor thrombotic microangiopathy (PTTM) is a rare clinicopathological condition characterized by tumor microemboli associated with severe clinical manifestations, including pulmonary hypertension (PH), right-side-heart failure, and sudden death [1]. It is well-known to be caused most frequently by gastric carcinoma (GC). Recent studies have reported that imatinib treatment could improve the survival and PH in patients with PTTM associated with GC [2]. However, there has been no report that Anlotinib was effective in PTTM caused by GC.

## Case report

**Fig. 1:**
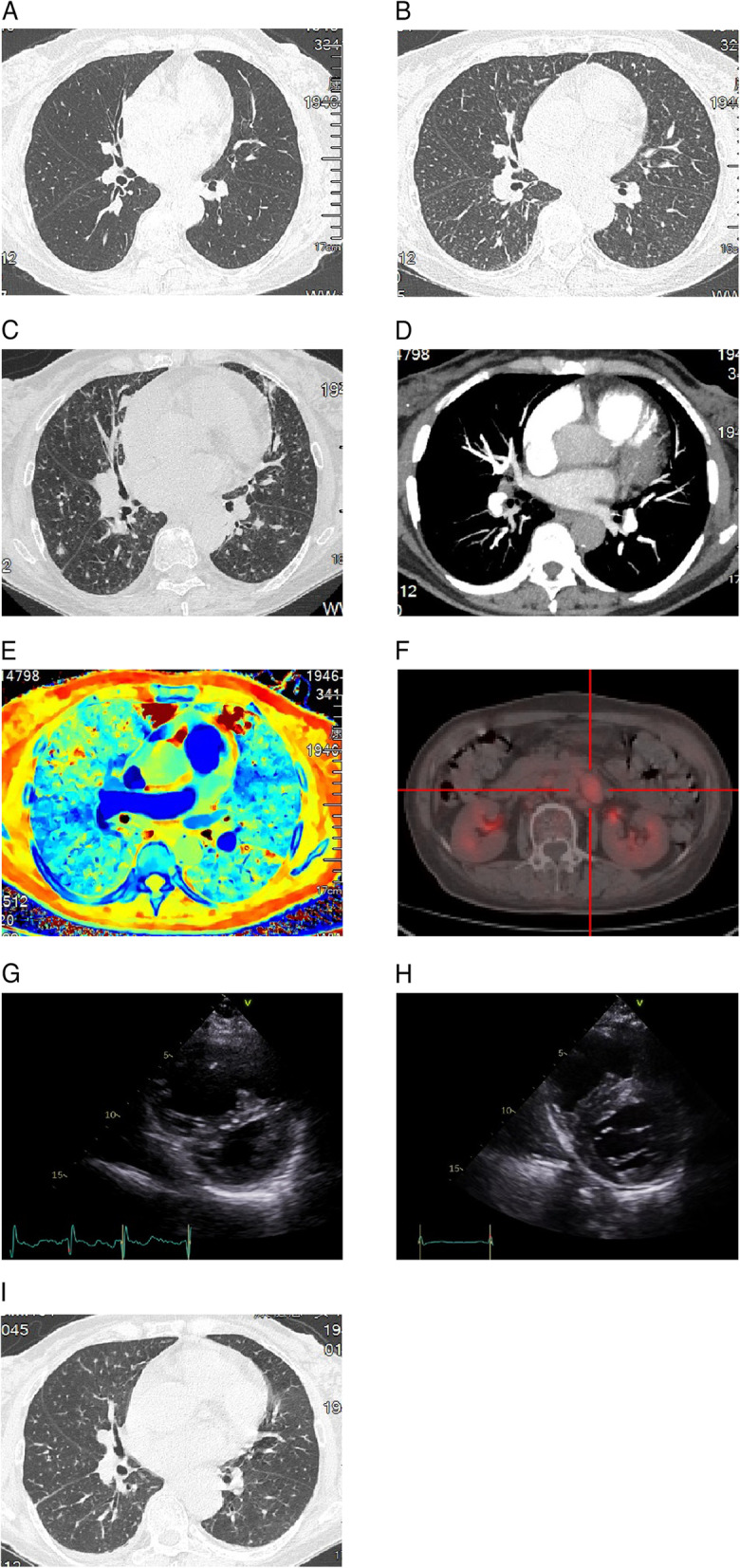
Chest radiography and electrocardiography. **A-C** CT showed increased diffusive nodules and thickening interlobular septal. **D** CTPA showed no specific findings of pulmonary embolism. **E** Impaired of pulmonary perfusion detected by dual-energy CT. **F** PET-CT showed increased FDG uptake in the abdominal cavity near left pararenal with maximum SUV of 4.26, and the maximum cross-sectional area was about 28 × 18 mm. **G** TTE revealed enlargement of right ventricle, and compressed D-shaped left ventricle. **H** Echocardiography showed no evidence of D-shaped left ventricle after treatment. **I** Indicating improved chest CT after treatment

A 77-year-old female patient experienced chronic cough and progressing dyspnea two months before the admission. Initial chest computed tomography (CT) showed no specific findings of infectious pneumonia, or interstitial pneumonia (Fig. 1A). Flowingly, transthoracic echocardiography exhibited right ventricular enlargement and significant PH (TRPG of 80 mmg). However, computed tomographic pulmonary angiography (CTPA) showed no specific findings of pulmonary embolism, instead of increased diffusive nodules and thickening interlobular septal (Fig. 1B-D). Accordingly, the blood coagulation tests showed a slightly hypercoagulative state (D-dimer of 19.38 μg/mL, and fibrin degradation products of 42.20 μg/mL). NT-proBNP level was also elevated (5748 pg/mL, normal 0–125 pg/mL). Additionally, no evidence of autoimmune disease was found.

The anti-PH therapy (macitentan, 10 mg/day) and Methylprednisolone (40 mg/day) started immediately, but the patient’s respiratory condition kept getting worse. It was suggested that PH might be secondary to other diseases. Considering it, the patient was performed with PET-CT. PET showed increased FDG uptake in the abdominal cavity near left pararenal, and the maximum SUV was 4.26. CT showed soft tissue mass in the corresponding location, and the maximum cross-sectional area was about 28 × 18 mm (Fig. 1F). And no abnormal FDG uptake was found in other sites.

**Fig. 2:**
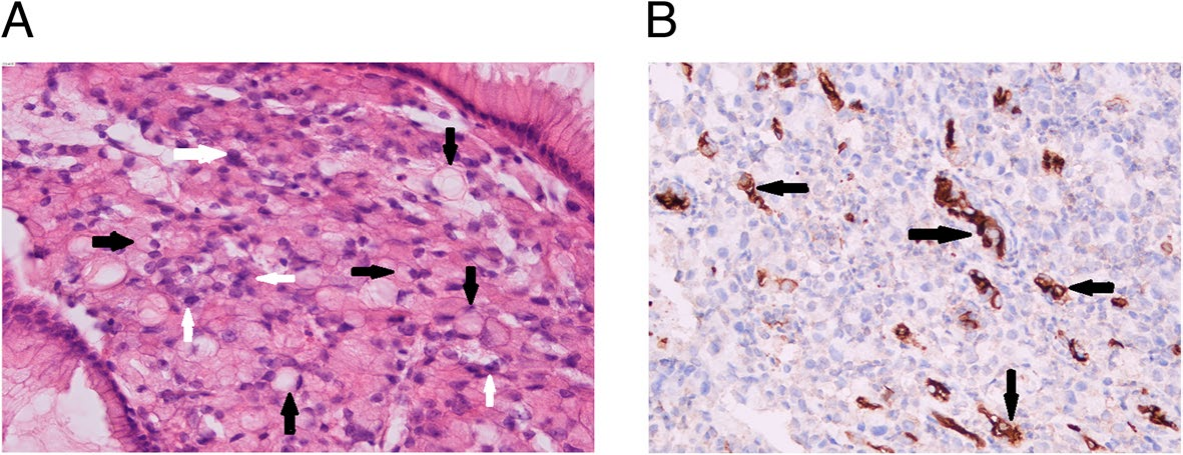
Histopathological analysis and Immunohistochemistry. **A** Hematoxylin–eosin staining analyzed the histopathological changes of biopsied gastric mucosa. Adenocarcinoma (white arrows), signet ring cell carcinoma (black arrows) (400 ×). **B** VEGF expression was analyzed by immunohistochemistry (Streptavidin-Perosidase assay, Anti-VEGF antibody, GeneTech, VG1; 400 ×). Most signet ring cell carcinoma cells were negative for VEGF, while some adenocarcinoma cells were positive (arrows)

Then, the patient was transferred to RICU for further pulmonary assessment and high-flow nasal oxygen (HFNO) therapy. In the next few days, CT-guided needle aspiration of abdominal mass and gastroscope were performed, which both suggested adenocarcinoma derived from gastric tissue composing of signet-ring cell carcinoma (Fig. 2A). Meanwhile, VEGF expression was analyzed by immunohistochemistry. Most signet ring cell carcinoma cells were negative for VEGF, while some adenocarcinoma cells were positive (Fig. 2B).

In context of pathological diagnosis of gastric adenocarcinoma, we clinically diagnosed PTTM based on the following clinical findings, (1) acute progression of respiratory failure with severe PH, (2) no specific CT findings of pulmonary embolism, (3) multiple subsegmental peripheral perfusion defect, (4) activation of coagulation cascade and formation of fibrin clots. Then, Anlotinib (8 mg once daily) was started.

**Fig. 3:**
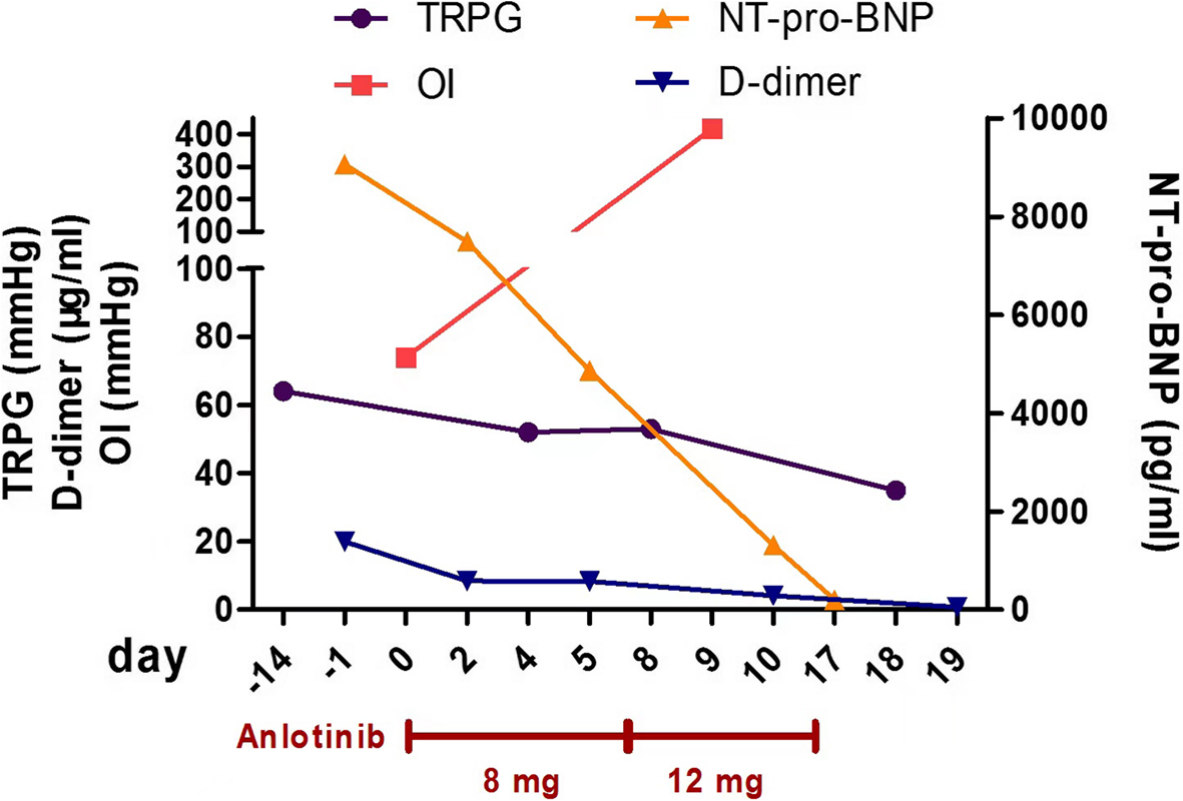
Clinical course of the patient with PTTM during admission. Timeline describing the patient’s treatment and clinical presentations are shown. Anlotinib improved pulmonary hypertension and hypoxia dramatically within 3 weeks

After the treatments, her respiratory condition was dramatically improved (Oxygenation index increased obviously) and the NYHA classification improved from IV to II. Serial TTE examination showed the disappearance of D-shaped left ventricle (Fig. 1G,H). Also, chest CT performed 2 weeks after oral anlotinib showed significant remission of diffusive pulmonary nodules (Fig. 1I). As shown in Fig. 3, clinical indicators of the patient during anlotinib therapy were serially present. In detailed, TRPG level was gradually reduced from 64 to 35 mmHg. D-dimmer was decreased from 20 to 0.81 μg/mL, and NT-proBNP from 9063 to 200.8 pg/mL.

We increased the dose of anlotinib to 12 mg/day after initial 1 week and continued macitentan (10 mg/day) and rivaroxaban (20 mg/day). She also received the combination of capecitabine and albumin-bound paclitaxel therapy.

## Discussion

Currently, it is difficult to obtain the pathological diagnosis of PTTM, leading to death in a few days to a few weeks typically without effective therapy. In term of pathogenesis of PTTM, cancer cells metastasize to the pulmonary vascular and adhere to the vascular endothelium. They not only cause mechanical obstruction of affected vessels, but also activate the coagulation cascade. These processes lead to diffuse narrowing of the pulmonary arteriolar system and increased vascular resistance, resulting in marked PH. Researches into possible mechanisms indicated the TF-VEGF system might be involved in the pathogenesis of PTTM [3]. Furthermore, osteopontin may be involved in fibrocellular proliferation and thrombus formation in PTTM, together with PDGF and VEGF [4]. Based on these, it was suggested that VEGF/PDGF could be proposed as a candidate target for therapy to PTTM. Accordingly, although a number of treatments have been attempted for patients diagnosed with or suspected of having PTTM, successful-treated cases of PTTM were mainly from imatinib therapy, which was a PDGF receptor inhibitor [2].

Considering of the active pulmonary arteriolar vascular endothelial cells during the development of PTTM, candidate molecules should be oriented to VEGF/PDGF, and to other facotrs related to fibrocellular proliferation and thrombus formation, in addition to treatment of the malignancy. Anlotinib was developed by Chia-tai Tianqing Pharmaceutical Co., Ltd. in China, which is a tyrosine kinase inhibitor that targets VEGFR, fibroblast growth factor receptor (FGFR), PDGFR, and c-kit. Preclinical studies have shown that anlotinib inhibits cell migration and the formation of capillary-like tubes induced by VEGF/PDGF-BB/FGF-2 in endothelial cells [5]. Another study revealed that anlotinib inhibits the activation of VEGFR2, PDGFRβ and FGFR1, as well as downstream ERK signaling. Among the known factors leading to PTTM, anlotinib may be the treatment which could block various factor-induced narrowing of the pulmonary arteriolar system and increased vascular resistance. To the best of our knowledge, this is the first case of PTTM caused by GC that anlotinib significantly improved PH and hypoxia.

## Conclusions

The present study revealed that a patient with GC developed PH and PTTM which could not be controlled by macitetan improved with the addition of anlotinib. As the pathologic diagnosis of PTTM is challenging and rarely achieved, treatment chance might lose due to rapid worsening of respiratory failure. We believe that our decision of clinical diagnosis of PTTM and starting anlotinib without waiting for pathological report in this patient can be reasonable. Clinical diagnosis and prompt initiation of anlotinib might be one of the strategies in patients with unstable PTTM. Improved understanding of the pathobiology of the disease process may help generate more efficacious therapeutic targets.

## Data Availability

All data generated or analysed during this study are included in this published article.

## Abbreviations

PTTM: Pulmonary Tumor Thrombotic Microangiopathy
PH: Pulmonary Hypertension
GC: Gastric Carcinoma
CT: Computerized Tomography
PET-CT: Positron Emission Tomography-Computed Tomography
FDG: Fluorodeoxyglucose
SUV: Standardized Uptake Value
NYHA: New York Heart Association
TRPG: Tricuspidvalve Regurgitation Pressure Gradient Biggest
OI: Oxygenation index
PDGF: Platelet Derived Growth Factor
TF-VEGF: Tissue Factor—Vascular Endethelial Growth Factor
FGF: Fibroblast Growth Factor
FGFR: Fibroblast Growth Factor Receptor
PDGFR: Platelet Derived Growth Factor Receptor
ERK: Extracellular Signal Regulated Kinase
